# Supplementary material for: Toward Overcoming Pyrethroid Resistance in Mosquito Control: The Role of Sodium Channel Blocker Insecticides
Source: Int J Mol Sci. 2023 Jun 19;24(12):10334. doi: 10.3390/ijms241210334 (PMC10299721; doi:10.3390/ijms241210334)
Supplement: Supplementary file 1 [file ijms-24-10334-s001.zip › ijms-2379986-supplementary.pdf]

# Toward overcoming pyrethroid resistance in mosquito control: the role of sodium channel blocker insecticides

Beata Niklas [1,*], Jakub Rydzewski [1], Bruno Lapied [2] and Wieslaw Nowak [1,*]

[1] Institute of Physics, Faculty of Physics, Astronomy and Informatics, Nicolaus Copernicus University, Grudziadzka 5, 87-100 Torun, Poland;
[2] University Angers, INRAE, SIFCIR, SFR QUASAV, F-49045 Angers, France
* E-mail: beata.niklas@doktorant.umk.pl; wiesiek@umk.pl

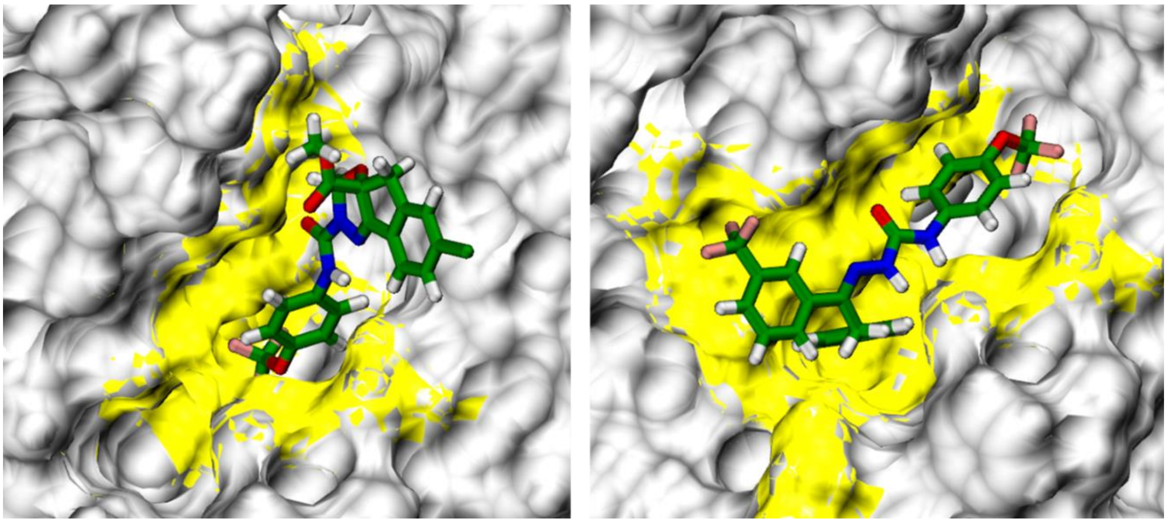

DCJW, SSF=-8.05 kcal/mol metaflumizone, SSF=-8.42 kcal/mol

Figure S1. Docking of DCJW (left) and metaflumizone (right) to the closed-state AgNav1 model. In their lowest energy poses (SSF, smina scoring function) ligands approach the DIII-DIV fenestration. Hydrophobic residues in a distance of 5 Å from each ligand are in yellow.

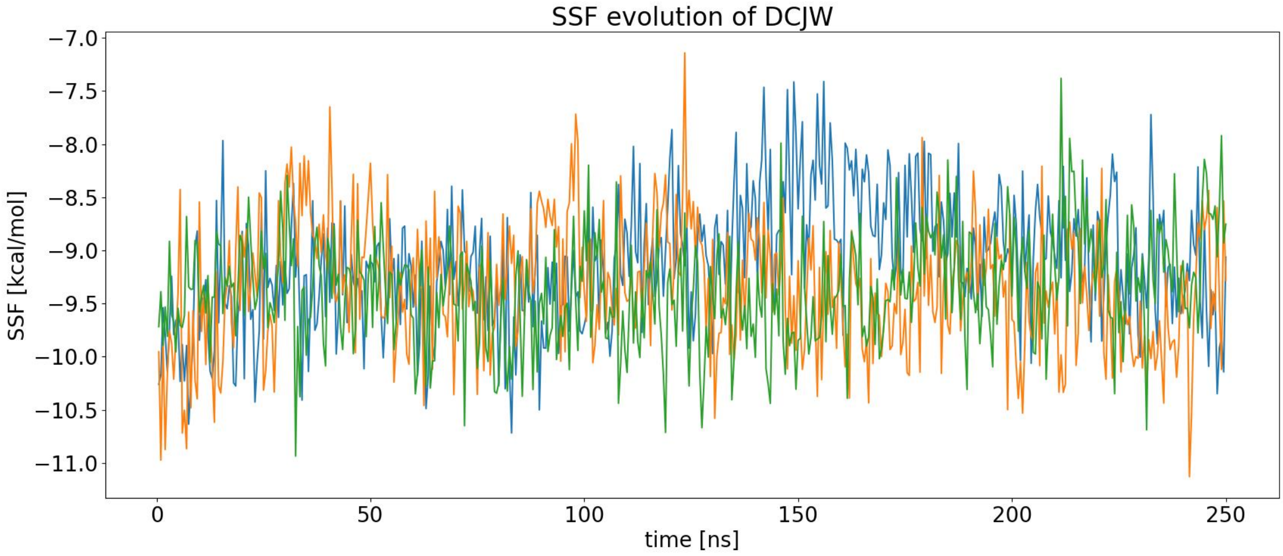


Figure S2. The values of smina scoring function (SSF) for DCJW bound in the inactivated-state AgNav1 model during three independent MD trajectories (orange, blue, and green).

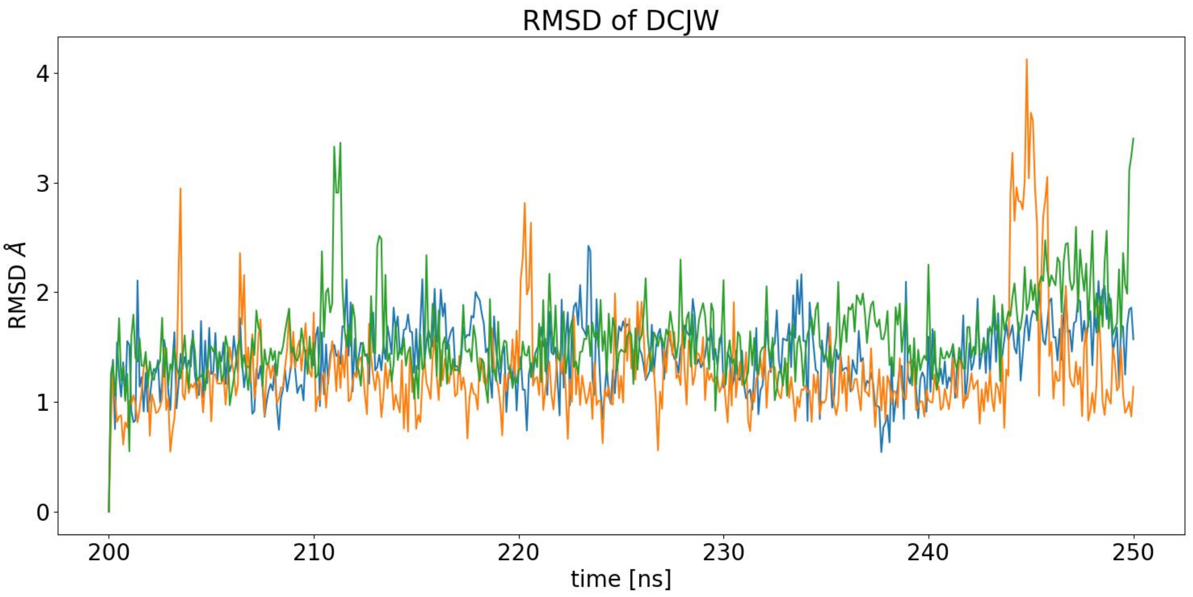


Figure S3. The root-mean-square deviation (RMSD) values over the last 50 ns from 250ns-long MD trajectories of DCJW-bound inactivated-state AgNav1 model.

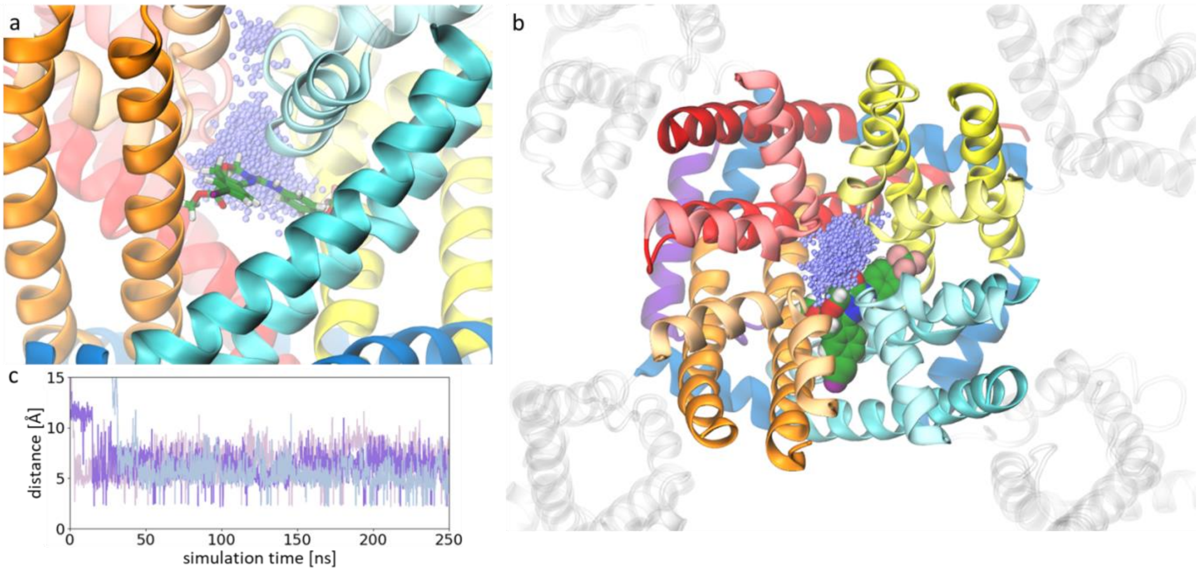


Figure S4. DCJW interaction with sodium ion. Side (a) and top (b) view of the inactivated-state mosquito VGSC with DCJW bound. The sodium ions positions found in the pore domain of the channel in three 250 ns MD trajectories are overlapped and presented as violet dots. The shortest distance between the ion and the carbonyl group of DCJW is shown in (c) where three shades of violet indicate three MD trajectories.

Table S1. Percentage of MD snapshots in which contact between DCJW and a given VGSC residue was found. Residues known to affect channel sensitivity to SCBIs are bolded.

| segment | residue | MD1 | MD2 | MD3 | mean |
|---|---|---|---|---|---|
| S6 DIV | **F1852** | 94 | 97 | 97 | 96 |
| S6 DIII | F1553 | 81 | 91 | 91 | 88 |
| DIII P | F1512 | 73 | 94 | 83 | 83 |
| DIII P | T1511 | 77 | 76 | 87 | 80 |
| S6 DIII | I1548 | 80 | 83 | 75 | 79 |
| S6 DIII | L1556 | 78 | 75 | 82 | 78 |
| DII P | C988 | 76 | 75 | 73 | 75 |
| S6 DII | **V1021** | 69 | 75 | 76 | 73 |
| DIII P | A1510 | 73 | 49 | 97 | 73 |
| S6 DIII | S1552 | 64 | 36 | 79 | 60 |
| DIII P | **F1507** | 58 | 73 | 22 | 51 |
| S6 DIII | I1549 | 47 | 50 | 51 | 50 |
| DII P | L987 | 47 | 33 | 58 | 46 |
| S6 DIV | L1848 | 27 | 90 | 12 | 43 |
| S6 DIII | **T1555** | 49 | 00 | 73 | 41 |
| S5 DIII | W1445 | 50 | 01 | 69 | 40 |
| S5 DIII | C1441 | 37 | 00 | 81 | 39 |
| S6 DIV | V1849 | 18 | 84 | 06 | 36 |
| DIV P | S1805 | 26 | 72 | 06 | 34 |
| S5 DIII | L1438 | 32 | 00 | 67 | 33 |
| S6 DIV | **V1855** | 10 | 33 | 01 | 15 |
| S6 DIV | **Y1859** | 11 | 25 | 00 | 12 |
| DII P | F984 | 19 | 09 | 02 | 10 |
| DIV P | **T1804** | 04 | 25 | 00 | 10 |

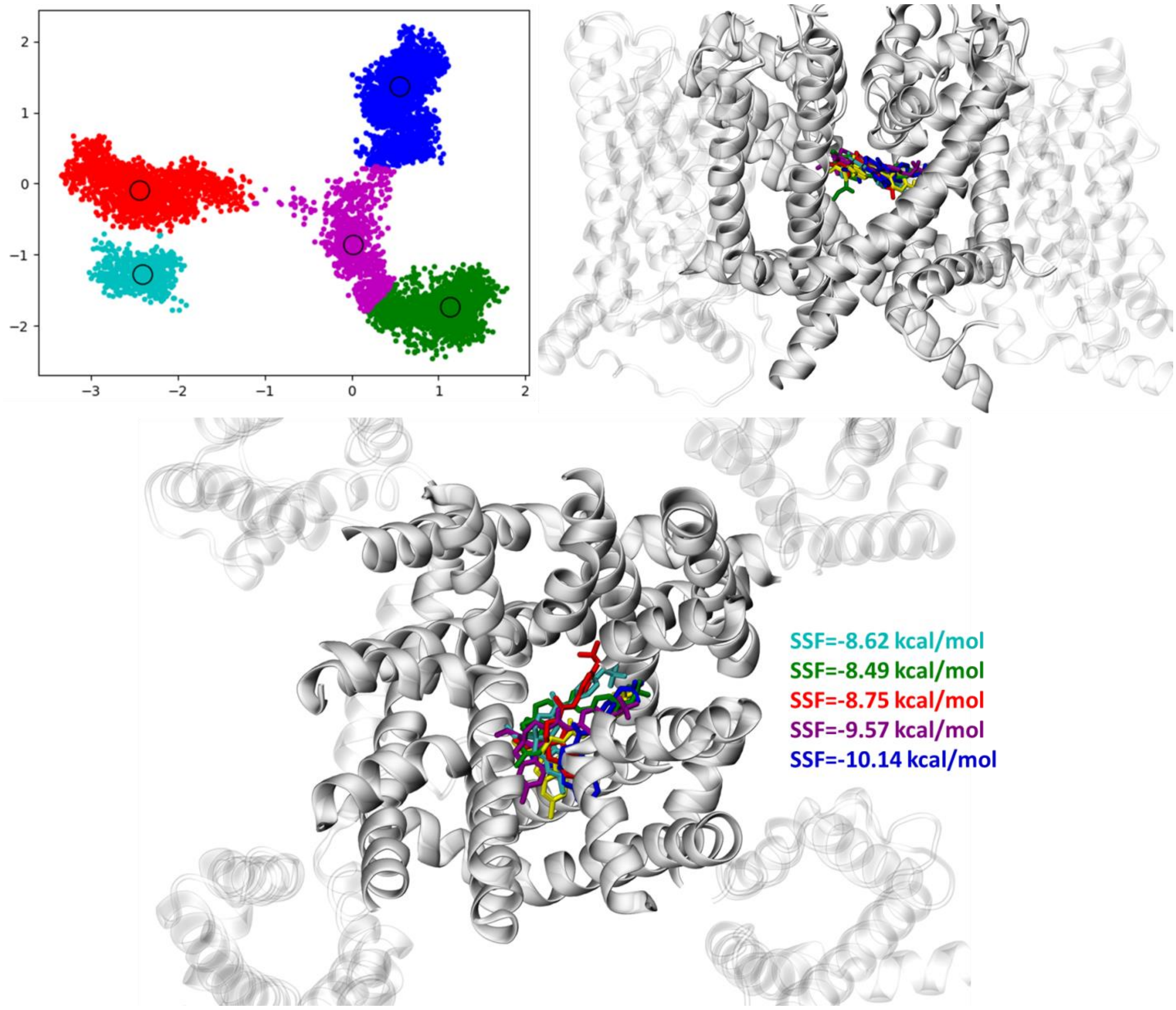


Figure S5. Docking of DCJW to the AgNav1 clusters from MD. The Principal Component Analysis (PCA) performed on the three repetitions of 250 ns MD simulation of the inactivated-state AgNav1 channel gave five clusters (upper left). Snapshot corresponding to the geometrical center of each cluster (marked in circles) was extracted and docking of DCJW has been run to find the poses of the ligand in the fluctuating conformations of a channel. The lowest energy positions of the ligand in each snapshot are shown in the side view (upper right) and top view (lower panel) with their smina scoring function (SSF) values. The position of the ligand found in docking to the static model is shown for comparison in yellow.

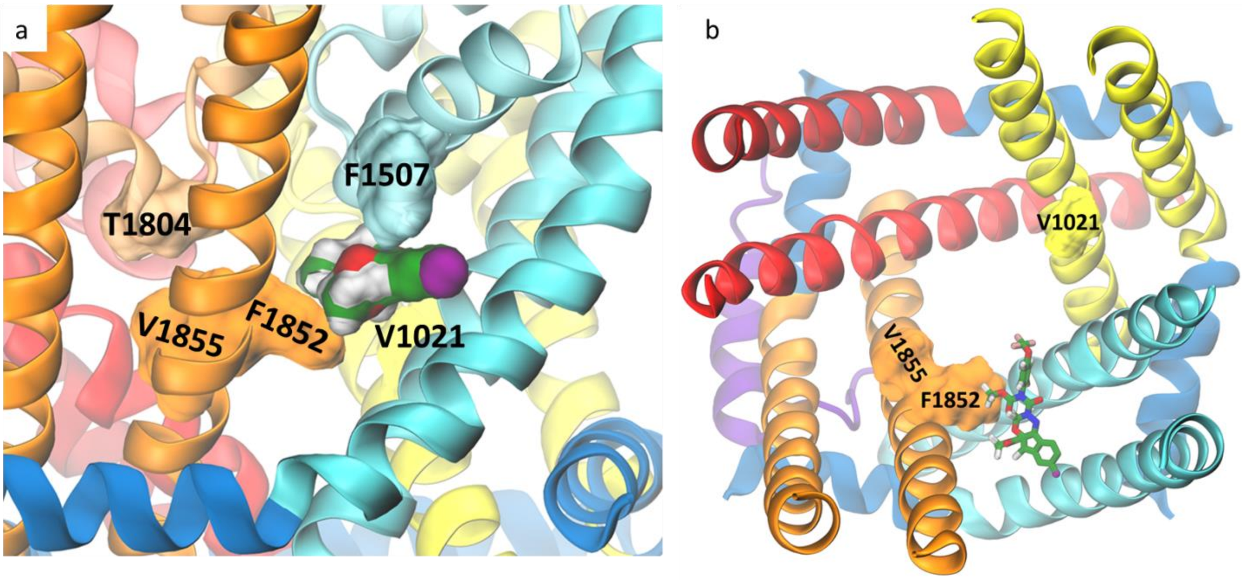


Figure S6. Docking of indoxacarb to the inactivated-state mosquito VGSC. Side view (a) and top view with the P-loops removed for clarity (b) are presented with the residues found to affect the channel sensitivity to blocker insecticides shown in the surface representation.

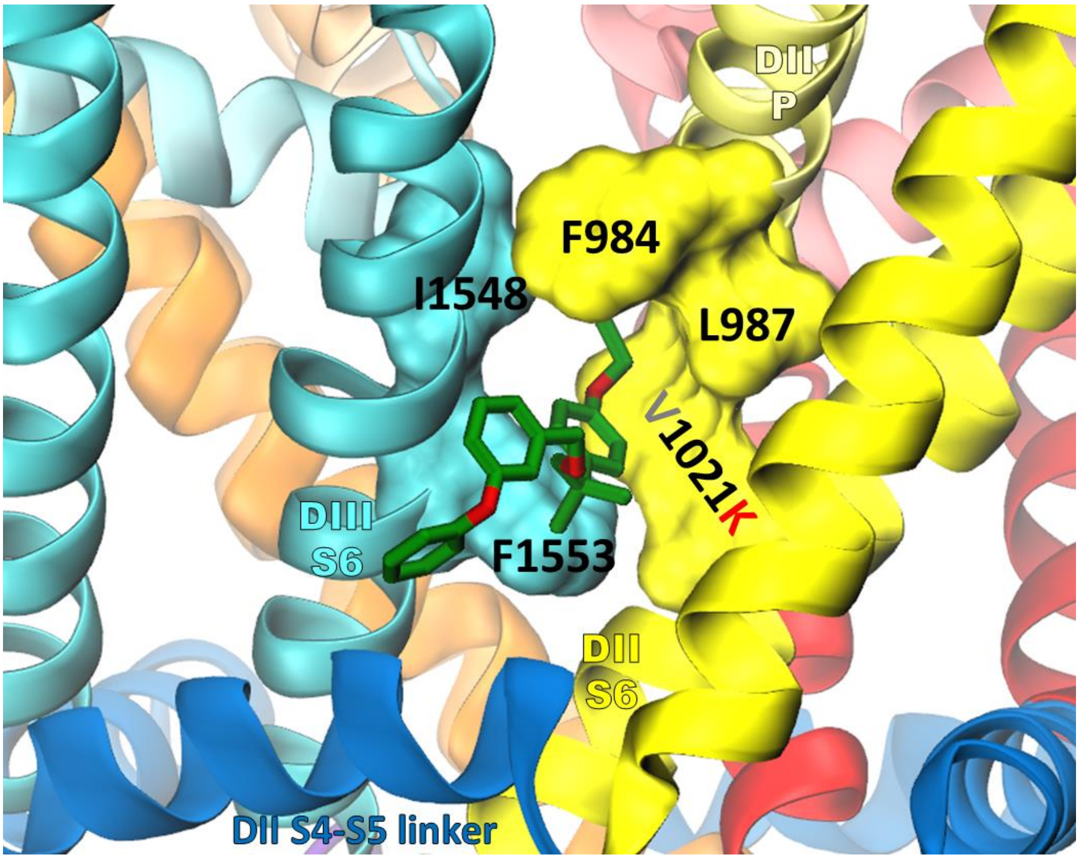


Figure S7. Etofenprox docking to the V1021K mutant of the mosquito sodium channel. Residues found to confer pyrethroid resistance (kdr) contributing to both etofenprox and SCBIs binding are shown in a surface representation.
